# Protective Role of Nuclear Factor-Erythroid 2-Related Factor 2 Against Radiation-Induced Lung Injury and Inflammation

**DOI:** 10.3389/fonc.2018.00542

**Published:** 2018-11-23

**Authors:** Xiaoli Tian, Feng Wang, Yuan Luo, Shijing Ma, Nannan Zhang, Yingming Sun, Chengcheng You, Guiliang Tang, Shuying Li, Yan Gong, Conghua Xie

**Affiliations:** ^1^Department of Radiation and Medical Oncology Zhongnan Hospital of Wuhan University, Wuhan, China; ^2^Hubei Key Laboratory of Tumor Biological Behaviors Zhongnan Hospital of Wuhan University, Wuhan, China; ^3^Department of Biological Repositories Zhongnan Hospital of Wuhan University, Wuhan, China; ^4^Hubei Cancer Clinical Study Center Zhongnan Hospital of Wuhan University, Wuhan, China

**Keywords:** radiation-induced lung injury, inflammation, Nrf2, oxidative damage, infiltration, cytokines

## Abstract

Radiation-induced lung injury (RILI) is one of the most common and fatal complications of thoracic radiotherapy. Inflammatory cell infiltration, imbalance of inflammatory cytokines, and oxidative damage were reported to be involved during RILI pathogenesis, especially in the early phase of RILI. Nuclear factor-erythroid 2-related factor 2 (Nrf2) is a key transcriptional regulator of antioxidative cascades, and regulates life span of mice after administration of thoracic irradiation. We investigated the effects of Nrf2 on RILI and inflammation using Nrf2-knockout, Nrf2-overexpression and wild-type mice with or without 15 Gy ionizing radiation to thorax. Our results showed that Nrf2 deficiency aggravated radiation-induced histopathological changes, macrophage and neutrophil infiltration, serum levels of pro-inflammatory cytokines (IL-6, MCP-1, IFN-γ, TNF, and IL-12p70), and the levels of peroxidation products in the mouse lung. Moreover, loss of Nrf2 reduced radiation-induced serum levels of anti-inflammatory cytokine, IL-10, and antioxidative proteins. Nrf2 overexpression significantly alleviated radiation-induced histopathological changes, macrophages and neutrophils infiltration, serum levels of pro-inflammatory cytokines, and the levels of peroxidation products in lung tissues. Nrf2 overexpression also increased the serum levels of IL-10 and antioxidative proteins. These results indicated that Nrf2 had a protective role against radiation-induced acute lung injury and inflammation, and that antioxidative therapy might be a promising treatment for RILI.

## Introduction

Radiation-induced lung injury (RILI) is one of the most common and fatal complications in radiotherapy for thoracic cancers, and limits the effective radiation dosage applied to eradicate tumors. Radiation-induced acute lung injury and inflammation involve a cascade of inflammatory events following ionizing radiation (IR) (1, 2). Infiltration of macrophages and neutrophils in lung tissues and high serum levels of inflammatory cytokines are recognized markers for RILI and inflammation (2–5). Until now, there is no effective prevention and treatment methods for RILI. Although steroids had been used to treat RILI, severe side effects remain a huge challenge.

IR induces cellular damage by direct deposit of energy and indirect oxidative damage via excessive reactive oxygen species (ROS), which were reported to be implicated in radiation-induced pulmonary injury, inflammation and fibrosis (6, 7). IR induces the accumulation of ROS, including hydroxyl radicals, hydrogen peroxide, and superoxide. ROS accumulation in lung tissues results in protein, lipid, and DNA damage, such as single strand breaks, double strand breaks, protein DNA crosslinks, as well as, other types of oxidative damage (8). These damages cause cell apoptosis and extracellular matrix remodeling, which were the secondary injury of IR. Nuclear factor-erythroid 2-related factor 2 (Nrf2) is a well-characterized and redox-sensitive transcriptional activator, whose activity is tightly controlled by cytoplasmic association with its inhibitor Keap1. Upon oxidative stress, Nrf2 dissociates from Keap1 and translocates into the nucleus to activate a series of antioxidant response elements (AREs), such as glutathione peroxidase (GPx), superoxide dismutase (SOD), catalase (CAT), heme oxygenase-1 (HO-1) (9). Recent studies indicated that Nrf2 reduced radiation-induced tissue injury by preventing alveolar type 2 cells loss after IR (10). Furthermore, Nrf2 deficiency reduced life span of mice after administration of thoracic irradiation (11). Therefore, we hypothesized that Nrf2 might be a protective factor against IR-induced acute lung injury and inflammation, and that activation of Nrf2 signaling pathway alleviated IR-induced lung injury. There were several studies reported that Nrf2 was involved in radiation-induced oxidative stress (12, 13), but the related mechanism study and the use of *in vivo* over-expressing animal model were lacking.

In this study, we explored the regulatory effects of Nrf2 in IR-induced lung injury and inflammation in the early phase of RILI using Nrf2-knockout and Nrf2-overespression mice. Our results indicated that Nrf2 protected against IR-induced lung injury and inflammation by reducing IR-induced oxidative damage in lung tissues. Our findings revealed the molecular mechanisms of Nrf2 regulation on RILI and might broaden our comprehensive understanding of RILI and improve the clinical outcomes.

## Materials and methods

### Mice and experimental design

Wild-type (Nfe2l2^+/+^) male C57BL/6 mice were obtained from Hubei Provincial Investigation Center for Animals (Wuhan, China) and Nrf2-knockout (Nfe2l2^−/−^) male mice were purchased from Jackson Laboratory (stock 017009). All mice were housed in a temperature-controlled and specific pathogen free environment. All experimental procedures and protocols were approved by the Medical Sciences Animal Care Committee of Zhongnan Hospital of Wuhan University. For loss-of-function study, 24 wild-type mice and 24 Nrf2-knockout mice were randomly divided into 4 groups: 1 non-instrumented control (NC) group (WT-CON and KO-CON), and 3 IR groups including IR1d group (WT-IR1d and KO-IR1d), IR7d group (WT-IR7d and KO-IR7d), IR14d group (WT-IR14d and KO-IR14d) group. Mice from the IR group received a single dose of 15 Gy thorax radiation. The mice were sacrificed and samples were collected on day 1, 7, and 14 after radiation. The validation of Nrf2-knockout mice was showed in Figure S1. For gain-of-function study, 20 wild-type mice were randomly divided into 4 groups: Lv-vector control group (injected with Lv-vector and no radiation), Lv-vector IR14d group (injected with Lv-vector and received radiation), Lv-Nfe2l2 control group (injected with Lv-Nfe2l2 to establish Nrf2 over-expression *in vivo* and no radiation) and Lv-Nfe2l2 IR14d group (injected with Lv-Nfe2l2 to establish Nrf2 over-expression *in vivo* and receive radiation). The mice were sacrificed and samples were collected on day 14 after radiation.

### Lentivirus delivery

Lentivirus carrying Nfe2l2 (Lv-Nfe2l2) was offered by Genechem (Shanghai, China). C57BL/6J mice were anesthetized and intratracheally injected with Lv-Nfe2l2 (5 × 10^6^ copies of lentivirus/mouse), and Lv-vector (5 × 10^6^ copies of lentivirus/mouse) was used as control. The *in vivo* transfection was performed 7 days before IR. The method of exposure of the mice to lentivirus has been previously described (14). The expression levels of Nrf2 mRNA and protein in lung tissue were detected by real-time quantitative polymerase chain reaction (qPCR) and western blot (Figures S2A,B). The efficiencies of lentiviral-mediated transfection were determined by fluorescent imaging of mice lung tissues (Figure S2C).

### Irradiation treatment

Mice were anesthetized and received a single dose of 15 Gy to the thorax using a small animal micro-CT irradiator X-RAD 225Cx (Precision X-ray Inc., North Branford, CT). The beam was 225-kV photon at a dose rate of 3.12 Gy/min. The source-surface distance (SSD) was 30 cm. The radiation field was 2 × 2 cm.

### Serum collection and tissue isolation

Mice were sacrificed at indicated times. Retro-ocular artery blood was collected and centrifuged at 5,000 rpm for 5 min and then at 3,000 rpm for another 5 min. The serum was collected and kept at −20°C for future analysis of cytokines. The upper left lung lobe from each mouse is fixed in 4% paraformaldehyde and then embedded in paraffin for histopathology analysis. The lower left and right lung lobes were snap-frozen in liquid nitrogen and used for RNA and protein isolation, measurement of hydroxyproline content, CAT activity and MDA levels.

### HE staining and immunohistochemistry

The left lung specimens were embedded in paraffin and cut into 4 μm-thick slices. Sections were deparaffinized in xylene and rehydrated in a graded ethanol series. Hematoxylin and eosin (HE) staining was performed using a HE staining kit (C0105, Beyotime, Hangzhou, China) according to the manufacturer's instructions. A pathologist who was unaware of the group assignment examined the sections. To measure the degree of lung injuries, 10 fields for 2 slides from each mouse were analyzed under a × 200 field of microscope. A scoring system of 0–4 was used for each section based on the degree of septal thickening, congestion, hemorrhage, edema, and leukocyte infiltration. The scoring criteria were as follows: 0, normal appearance; 1, light; 2, moderate; 3, strong; and 4, intense and widespread abnormality. The mean value of each mouse was used for statistical analysis. Immunohistochemical staining of F4/80, MPO, 8-OHdG, and 4-HNE were performed using an UltraSensitive™ SP Kit (KIT-9710, Maxim Bio, Fuzhou, China) following the standard protocol. The information of primary antibodies is shown as follows: anti-8-OHdG (ab62623, 1:1000, Abcam, UK), anti-4-HNE (ab46545, 1:200, Abcam, UK), anti-MPO (ab188211, 1:8000, Abcam, UK), and anti-F4/80 (GB11027, 1:1000, Servicebio, China). As negative controls for immunohistochemistry analysis, sections were incubated with non-immune serum instead of the primary antibody. Images were then analyzed with Image-Pro Plus 5.1. software (Media Cybernetics, Inc., Rockville, MD, USA). Immunoactivity of F4/80, MPO, and 8-OHdG were quantified with the percent of positive cells, immunoactivity of 4-HNE was quantified with the IOD and mean density (MD) = IOD/area.

### Measurement of serum cytokines

A BD Cytometric Bead Array (CBA) Mouse Inflammation Kit (552364, BD Biosciences, San Diego, CA) was used to measure interleukin-6 (IL-6), interleukin-10 (IL-10), monocyte chemoattractant protein-1 (MCP-1), interferon-γ (IFN-γ), tumor Necrosis Factor (TNF), and interleukin-12p70 (IL-12p70) levels in mice serum according to the manufacturer's instructions.

### Western blot analysis

Lung tissues were homogenized and incubated in lysis buffer containing a protease inhibitor cocktail. The protein concentrations were measured using a BCA kit (P0012, Beyotime, Hangzhou, China), and proteins were then denatured at 100°C for 5 min. Proteins were loaded onto a 10% SDS-PAGE gel and transferred to a polyvinylidene fluoride (PVDF) membrane. Blots were blocked at room temperature for 1 h and then incubated with primary antibodies at 4°C overnight. The information of primary antibodies is shown as follows: anti-β-actin (ab8227, 1:2000, Abcam, UK), anti-GAPDH (10494-1-AP, 1:2,000, Proteintech, China), anti-Nrf2 (ab137550, 1:500, Abcam, UK), anti-SOD2(06984, 1:500, Millipore, USA), anti-Glutathione Peroxidase 1 (GPX-1, 1:500, ab22604, Abcam, UK). After washing, blots were incubated with fluorescence-labeled secondary antibodies (IRDye700 and IRDye800, goat anti-mouse/rabbit, 1:10,000, Licor, USA). Signals were detected with an Odyssey infrared imaging system (Licor, USA).

### Measurement of CAT activity and MDA levels

Total proteins were extracted from mice lung tissues using RIPA buffer containing PMSF. After protein concentrations measurement using a BCA assay kit (P0012, Beyotime, Hangzhou, China), catalase (CAT) activity and malondialdehyde (MDA) levels in lung tissue was measured by a CAT assay kit (A007-1, Jiancheng Biological Institution, China) and a MDA kit (A003-1, Jiancheng Biological Institution, China) according to the manufacturer's instructions.

### Statistical analysis

Statistical analysis was performed using SPSS 19.0 (IBM Corporation, Armonk, NY, USA). Results were presented as mean ± SD. Multigroup comparisons were carried out by one-way analysis of variance (ANOVA) test. Differences between 2 groups were determined using Dunnet *t*-test. Multiple means were compared by Tukey's test. *P* < 0.05 were considered statistically significant.

## Results

### Nrf2 knockout aggravates radiation-induced lung injury

**Figure 1:**
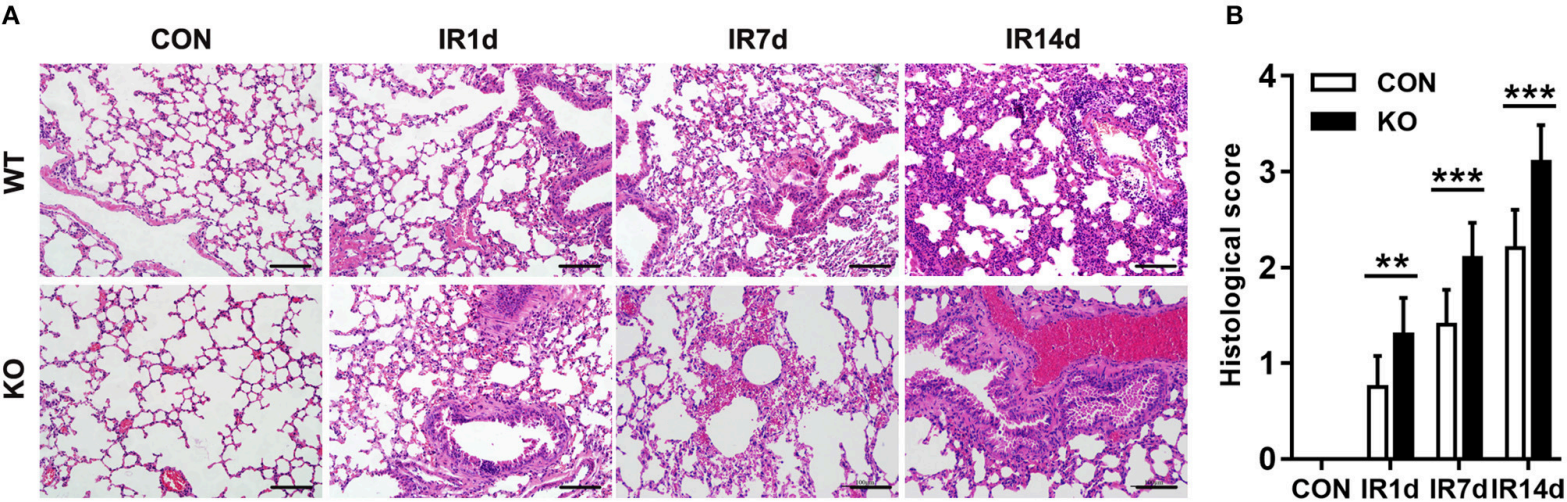
Nrf2 knockout aggravates radiation-induced lung injury. **(A)** HE staining was used to evaluate IR-induced structural destruction of the lung tissues. Scale bar: 100 μm. The representative figures of 3 independent experiments were presented here. **(B)** Histology scores of HE staning. *n* = 6. **P < 0.01; ***P < 0.001.

After 15 Gy local irradiation, increased alveolar septal thickness, structure damage, hemorrhage and inflammatory cell infiltration were observed in the sections of lung tissues. These pathologic changes of radiation-induced acute lung injury were worse in the Nrf2-knockout mice compared with the wild type controls (Figure 1).

### Loss of nrf2 increases radiation-induced infiltration of macrophages and neutrophils in lung tissues

**Figure 2:**
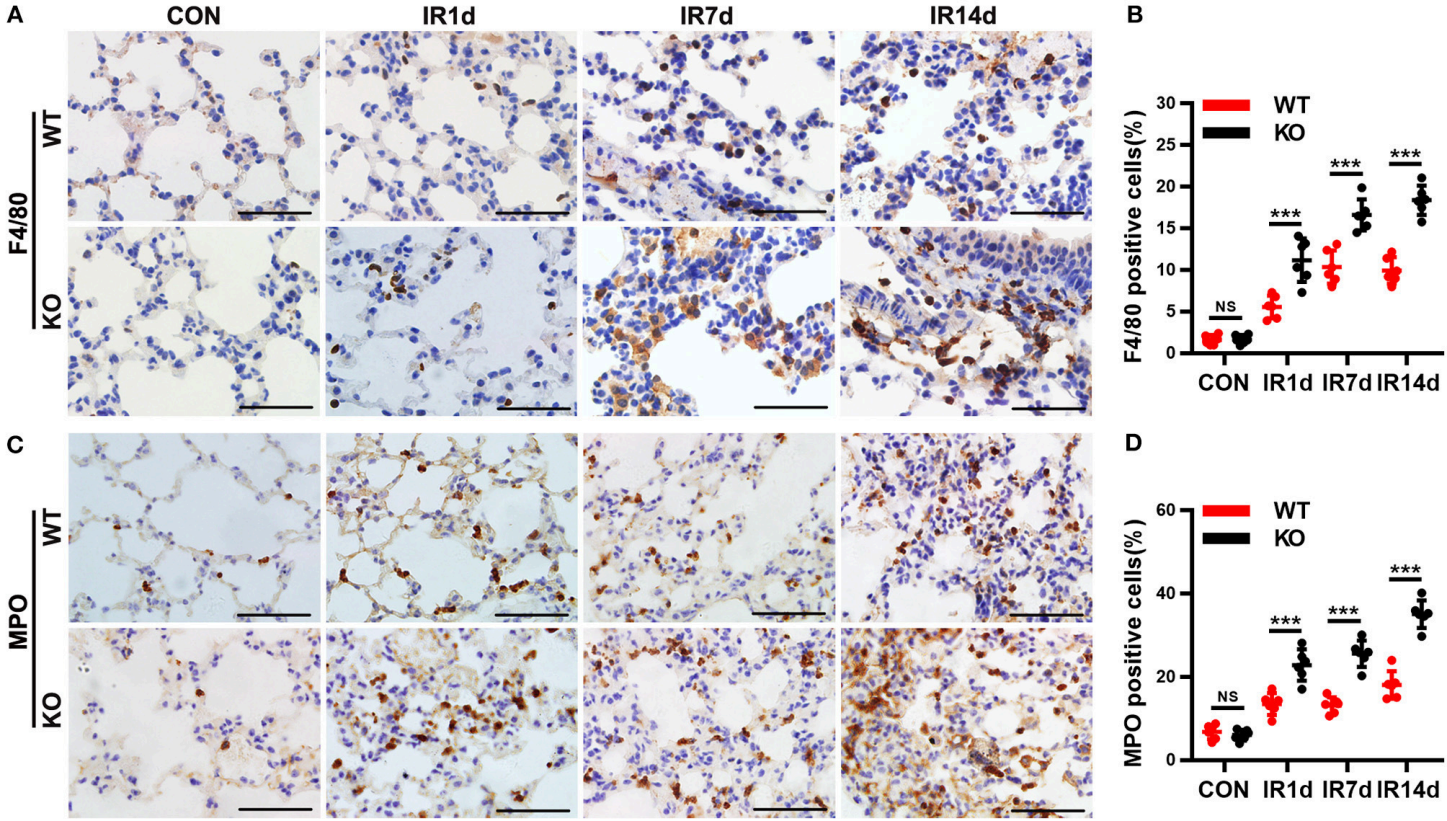
Nrf2 deficiency exacerbates radiation-induced infiltration of macrophages and neutrophils in lung tissues. **(A,B)** Immunohistochemistry staining of F4/80, a biomarker of macrophages, in lung tissues from the 8 groups of mice. Nrf2 knockout increased radiation-induced infiltration of macrophages. Scale bar: 50 μm. **(C,D)** Immunohistochemistry staining of MPO, a biomarker of neutrophils, in lung tissues of mice. Loss of Nrf2 contributed to radiation-induced infiltration of neutrophils. Scale bar: 50 μm. *n* = 6. ^NS^Represents no statistical difference (*P* > 0.05). ***P < 0.001. The data was shown as *mean* ± *SD*. The representative figures of 3 independent experiments were presented here.

Infiltration of macrophages and neutrophils in lung tissues is one of the most important pathological characteristics of RILI and inflammation. F4/80 and MPO are the biomarkers of macrophages and neutrophils. Our data indicated that infiltration of macrophages (Figures 2A,B) and neutrophils (Figures 2C,D) were significantly increased after IR in a time-dependent manner in both wild-type mice and Nrf2-knockout mice. Moreover, both F4/80 (Figures 2A,B) and MPO (Figures 2C,D) positive cell rates in lung tissues of Nrf2-knockout mice were significantly higher than those in wild-type controls. These results suggested that Nrf2 deficiency exacerbated radiation-induced infiltration of macrophages and neutrophils in the lung of mice.

### Nrf2 deficiency exacerbates radiation-induced imbalance of serum inflammatory cytokines

**Figure 3:**
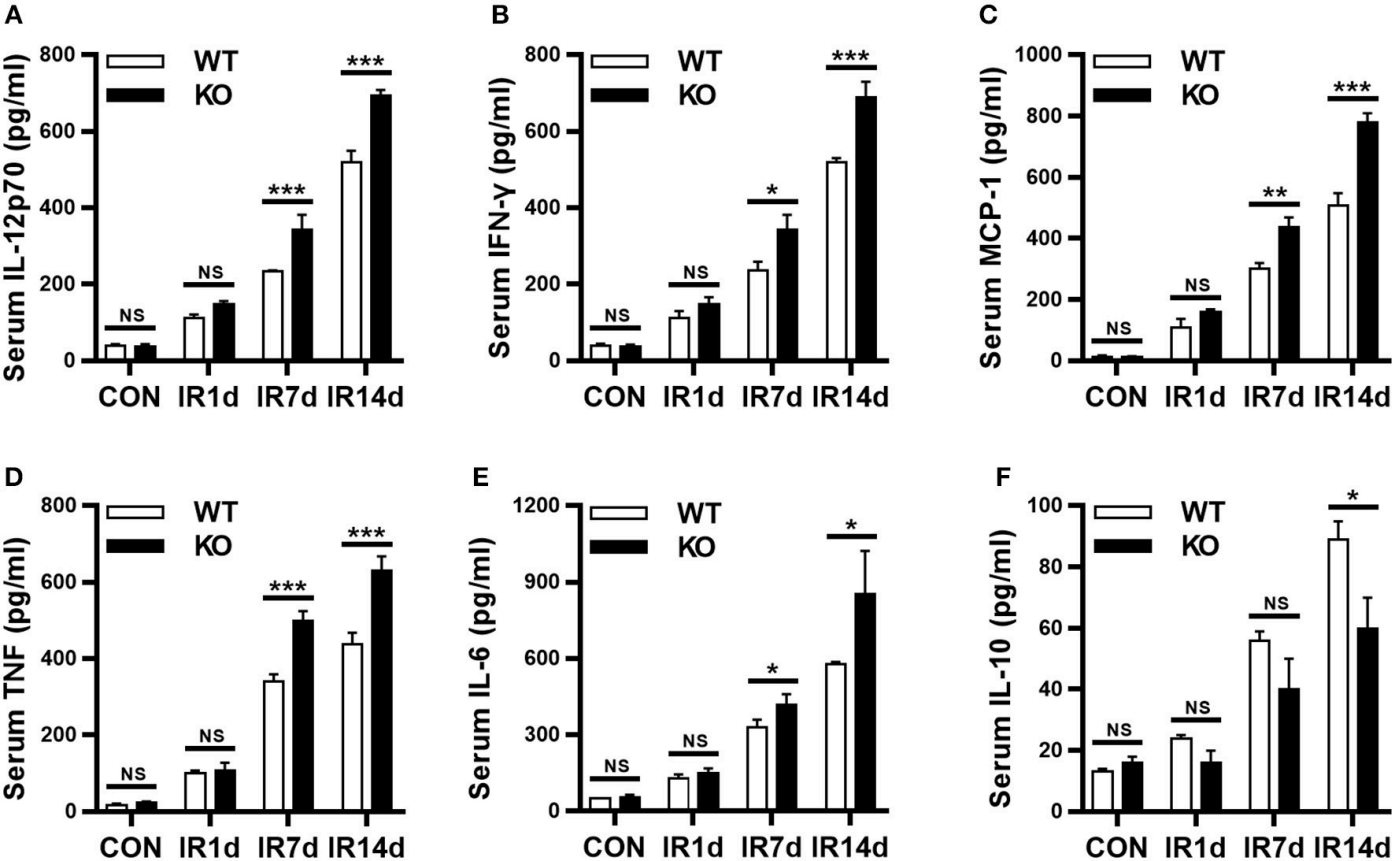
Loss of Nrf2 aggravates radiation-induced imbalance of serum inflammatory cytokines. BD CBA Mouse Inflammation Kit was used to measure the serum levels of inflammatory cytokines at different time points. Nrf2 knockout increased radiation-induced serum levels of IL-12p70 **(A)**, IFN-γ **(B)**, MCP-1 **(C)**, TNF **(D)**, and IL-6 **(E)**. Nrf2 knockout inhibited radiation-induced serum levels of IL-10 **(F)**. *n* = 6. ^NS^Represents no statistical difference (*P* > 0.05). *P < 0.05; **P < 0.01; ***P < 0.001. Every experiment was repeated for 3 times and the data was shown as *mean* ± *SD*.

Serum inflammatory cytokines are often used to evaluate RILI and inflammation. IL-12p70, IFN-γ, MCP-1, TNF, and IL-6 are in the pro-inflammatory profile, and IL-10 is in the anti-inflammatory profile. Our results showed that IR significantly increased the serum levels of IL-12p70, IFN-γ, MCP-1, TNF and IL-6, as well as, IL-10, in both wild-type and Nrf2-knockout mice in a time-dependent manner (Figures 3A–F). In addition, on day 7 and 14 after IR, the serum levels of IL-12p70, IFN-γ, MCP-1, TNF, and IL-6 in Nrf2-knockout mice were significantly higher than those in wild-type mice (Figures 3A–E). On the contrary, the serum levels of IL-10 in Nrf2-knockout mice were significantly lower than those in wild-type mice on day 14 after IR (Figure 3F). These results indicated that Nrf2 deficiency aggravated radiation-induced imbalance of serum pro- and anti-inflammatory cytokines.

### Nrf2 knockout worsens radiation-induced oxidative damage in lung tissues

**Figure 4:**
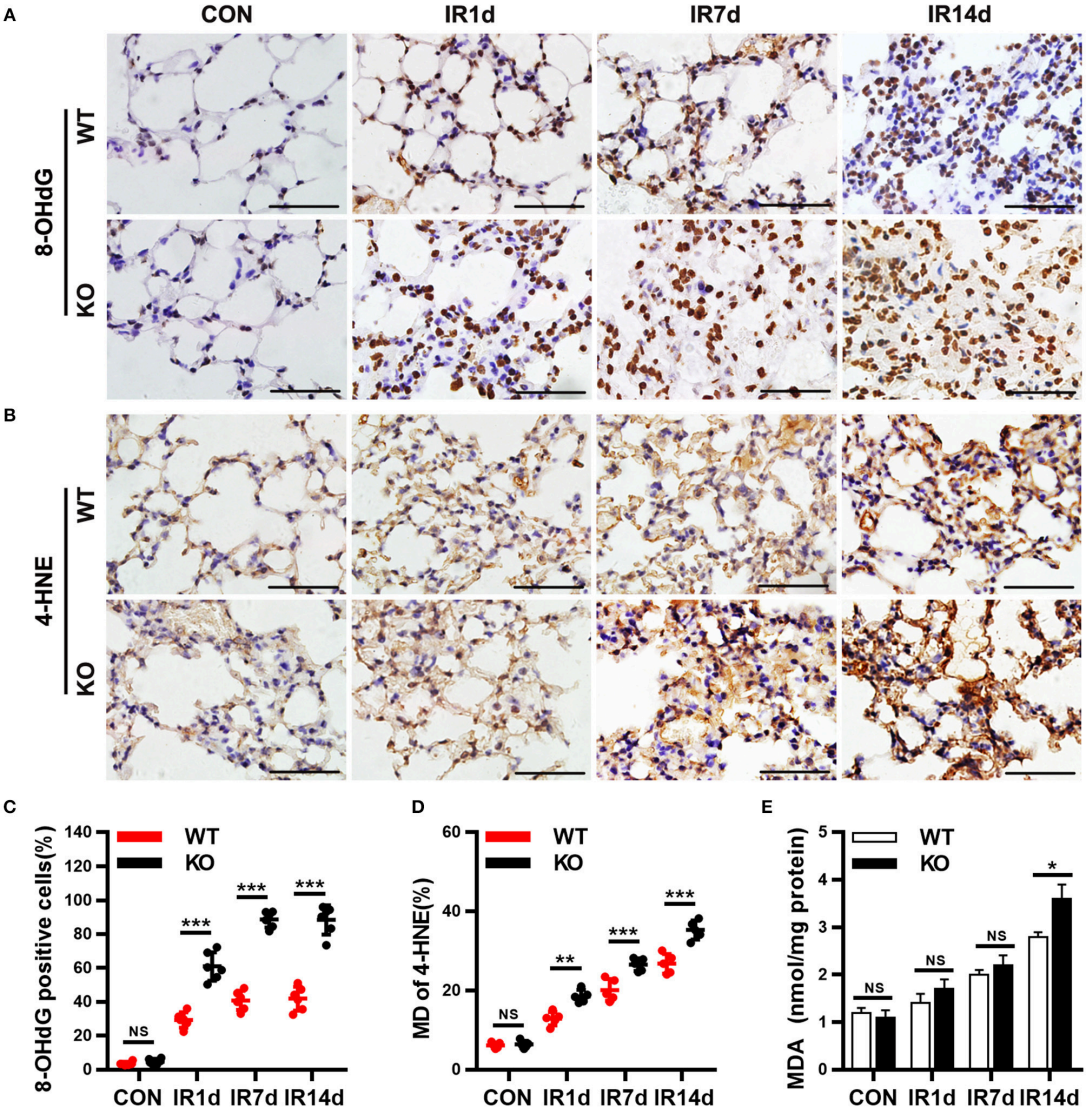
Nrf2 knockout worsens radiation-induced oxidative damage of lung tissues. **(A,C)** Nrf2 deficiency increased radiation induced 8-OHdG (a biomarker of DNA peroxidation product) positive cells in lung tissues. **(B,D)** Loss of Nrf2 increased 4-HNE (a biomarker of lipid peroxidation product) positive cells in lung tissues. **(E)** Nrf2 knockout upregulated the levels of MDA, another biomarker of lipid peroxidation product, in lung tissues. Scale bar: 50 μm. *n* = 6. ^NS^Represents no statistical difference (*P* > 0.05). *P < 0.05; **P < 0.01; ***P < 0.001. Every experiment was repeated for 3 times. The data was shown as *mean* ± *SD*, and the representative figures were presented here.

8-OHdG, 4-HNE, and MDA are the most commonly used markers of oxidative damage. In this study, the results showed that IR significantly elevated levels of 8-OHdG, 4-HNE, and MDA in a time-dependent manner in both Nrf2-knockout and wild-type mice (Figures 4A–E). The levels of 8-OHdG and 4-HNE in lung tissues of Nrf2-knockout mice were significantly higher than those in wild-type mice (Figures 4A–D). Similarly, on day 14 after IR, the levels of MDA in lung tissues of Nrf2-knockout mice was significantly higher than those in wild-type mice (Figure 4E). These results indicated that Nrf2 knockout exacerbated radiation-induced oxidative damage in lung tissues of mice.

### Nrf2 deficiency reduces radiation-induced antioxidant in lung tissues

**Figure 5:**
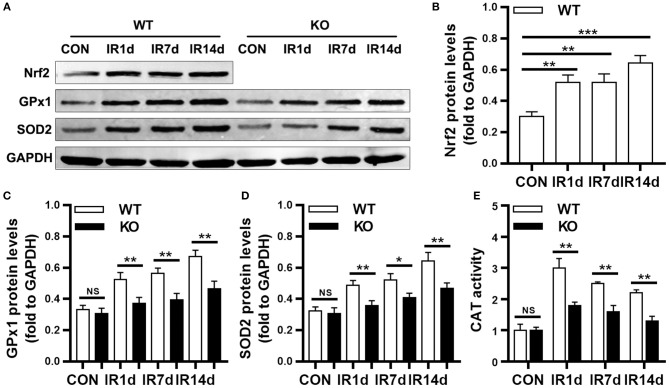
Nrf2 knockout suppressed radiation-induced antioxidative proteins. **(A–D)** Nrf2 deficiency inhibitd radiation induced Nrf2, GPx1, and SOD2 in lung tissues as meaured by Western blot. **(E)** Nrf2 knockout reduced radiation-induced activation of CAT. *n* = 6. ^NS^Represents no statistical difference (*P* > 0.05). *P < 0.05; **P < 0.01; ***P < 0.001. Every experiment was repeated for 3 times. The data was shown as *mean* ± *SD* and the representative figures were presented here.

As shown in Figure 5A, the protein levels of Nrf2 in wild-type mice were significantly up-regulated after IR. Similarly, the protein levels of GPx1, SOD2 and the activity of CAT were significantly increased after IR in both Nrf2-knockout mice and wild-type mice (Figures 5A–E). The protein levels of GPx1, SOD2 and the activity of CAT in lung tissues of Nrf2-knockout mice were significantly lower than those in wild-type mice (Figures 5A–E). These results suggested that loss of Nrf2 suppressed radiation-induced antioxidative pathway in lung tissues of mice.

### Nrf2 overexpression mitigates radiation-induced lung injury and inflammation

**Figure 6:**
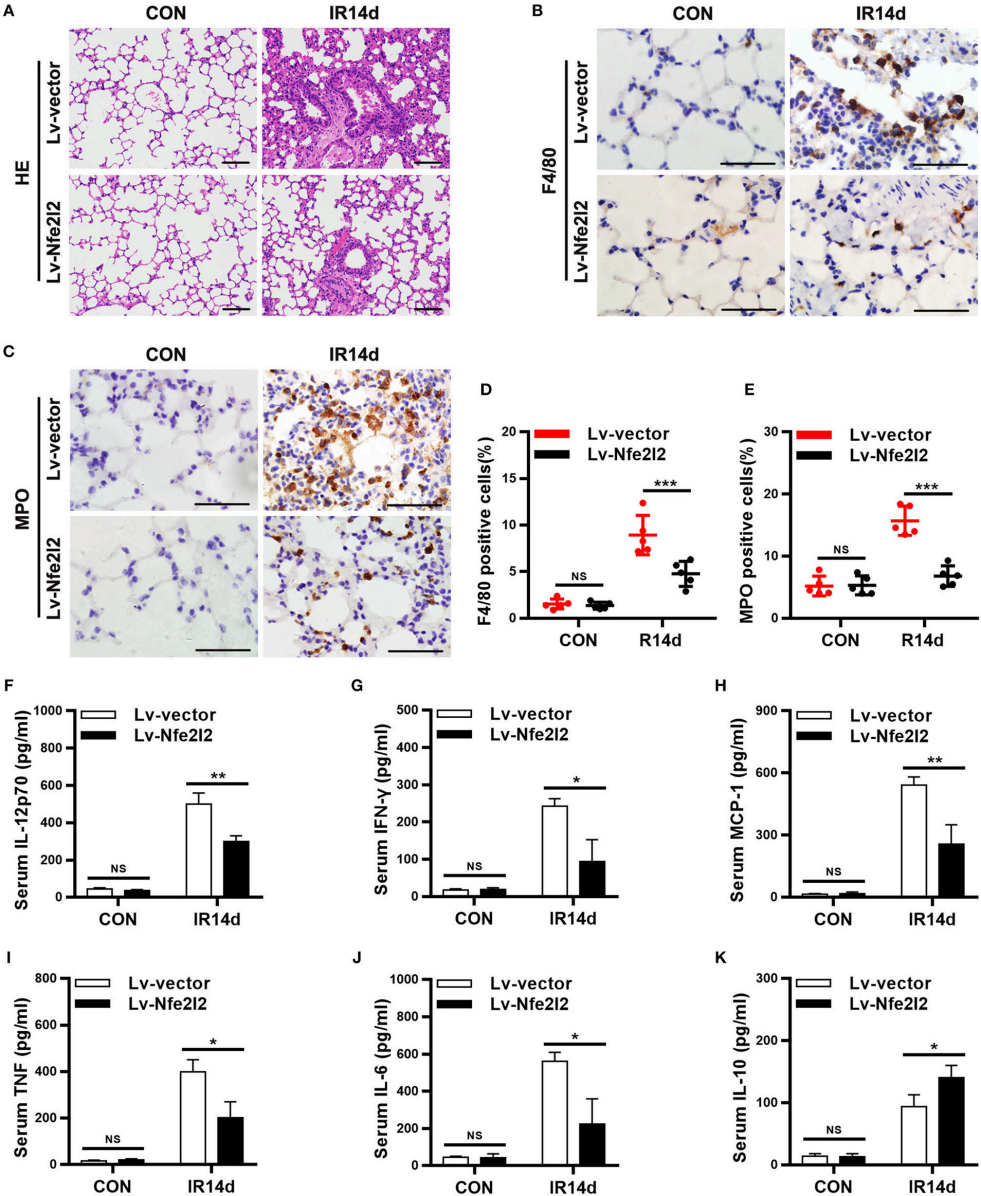
Nrf2 overexpression alleviates radiation-induced lung injury and inflammation. **(A)** Nrf2 overexpression attenuated radiation-induced lung injury as measured by HE staining. Scale bar: 100 μm. **(B,D)** Nrf2 overexpression decreased radiation-induced infiltration of macrophages as measured by immunohistochemistry staining. Scale bar: 50 μm. **(C,E)** Excess Nrf2 inhibited radiation-induced infiltration of neutrophils. Scale bar: 50 μm. **(F–K)** Nrf2 overexpression downregulated radiation-induced serum levels of pro-inflammatory cytokines (IL-12p70, IFN-γ, MCP-1, TNF, and IL-6) and increased radiation-induced serum levels of anti-inflammatory cytokines (IL-10). *n* = 5. ^NS^Represents no statistical difference (*P* > 0.05). *P < 0.05; **P < 0.01; ***P < 0.001. Every experiment was repeated for 3 times. The data was shown as *mean* ± *SD* and he representative figures were presented here.

In order to further confirm the important role of Nrf2 in RILI and inflammation, a Nrf2-overexpressing mice model was established via *in vivo* transfection. The results of HE staining showed that the alveolar septal thickness, structure damage, and hemorrhage were alleviated in lung tissues of Nrf2-overexpression mice compared with vector-control mice on day 14 after IR (Figure 6A; Figure S3). The infiltration of macrophages and neutrophils in lung tissues of Nrf2-overexpressing mice were significantly lower than those in vector-control group (Figures 6B–E). Similarly, Exogenous Nrf2 significantly reduced IR-induced serum levels of IL-12p70, IL-6, MCP-1, IFN-γ, and TNF (Figures 6F–J). In addition, the serum levels of IL-10 in Nrf2-overexpressing group was higher than those in the vector-control group (Figure 6K). Taking together, excess Nrf2 mitigated RILI and inflammation in mice.

### Nrf2 overexpression alleviates radiation-induced oxidative damage in lung tissues

**Figure 7:**
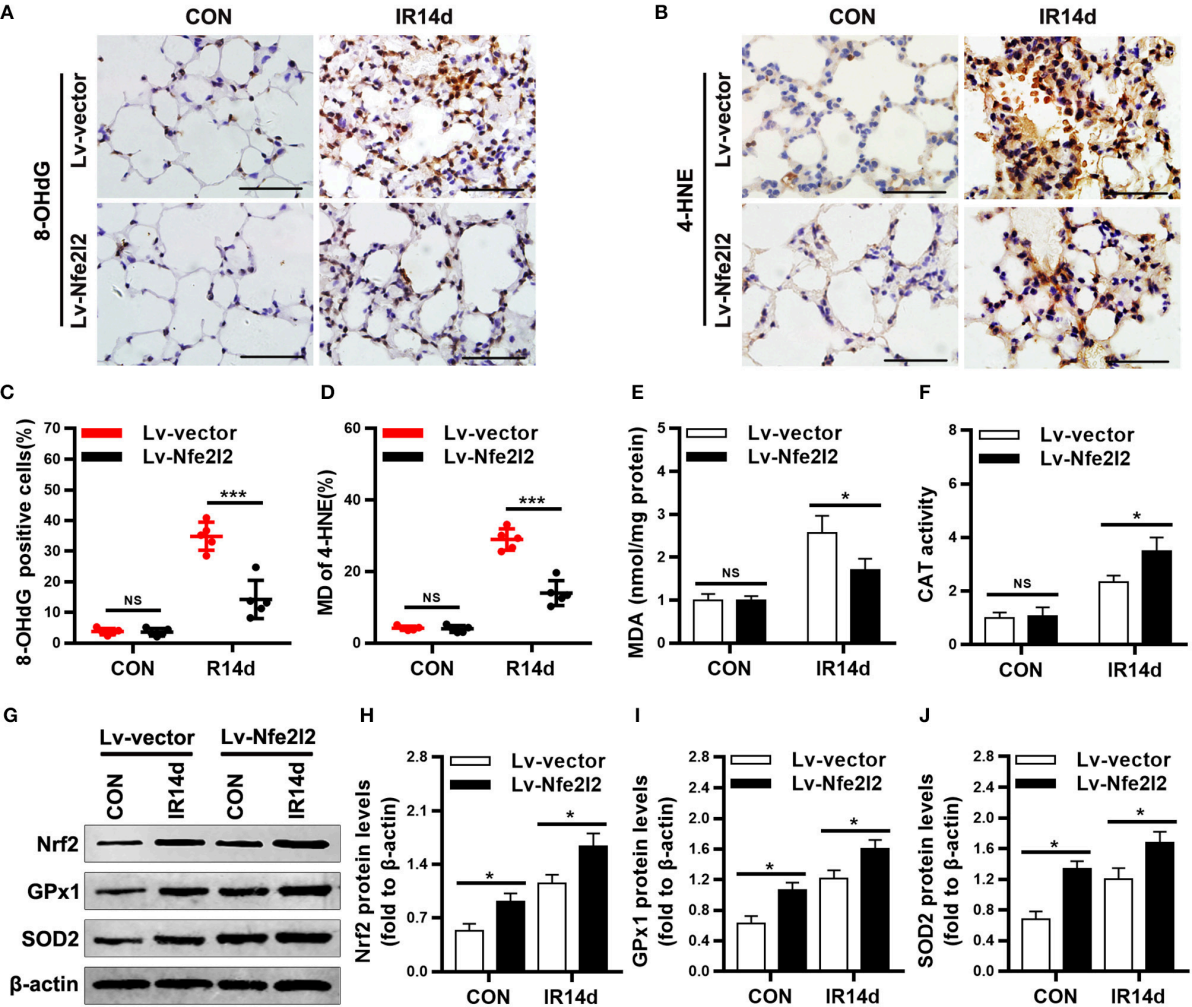
Excess Nrf2 attenuates radiation-induced oxidative damage in lung tissues. **(A,C)** Nrf2 overexpression decreased 8-OHdG positive cells in lung tissues as measured by immunohistochemistry staining. **(B,D)** Nrf2 overexpression decreased 4-HNE positive cells in lung tissues. **(E)** Levels of MDA in lung tissues were lower in Nrf2-overexpression mice compared with vector-control mice. **(F)** CAT activity in lung tissues was higer in Nrf2-overexpression mice compared with vector-control mice. **(G–J)** Excess Nrf2 upregulated the protein levels of Nrf2, GPx1, and SOD2 in lung tissues as measured by Western blot. Scale bar: 50 μm. *n* = 5. ^NS^Represents no statistical difference (*P* > 0.05). *P < 0.05; ***P < 0.001. Every experiment was repeated for 3 times. The data was shown as *mean* ±*SD* and the representative figures were presented here.

As shown in Figures 7A–E, the levels of 8-OHdG, 4-HNE, and MDA in lung tissues of Nrf2-overexpressing group were significantly lower than those in the vector-control group. On the contrary, the protein levels of Nrf2, GPx1, SOD2, and activity of CAT were significantly increased in lung tissues of Nrf2-overexpression group than those in vector-control group (Figures 7F–J).

## Discussion

RILI is one of the most common complications in the radiotherapy of chest tumor. IR-induced acute lung injury and inflammation characterized the early phase of RILI, which will progress to lung fibrosis. Lung fibrosis is a progressive and fatal disease which causes severe impairments of respiratory system. Till now, there is no available therapeutical strategy for RILI, and the underlying mechanism is still unclear. In this study, we found that Nrf2 deficiency aggravated the IR-induced acute lung injury, infiltration of microphages and neutrophils, imbalance of serum inflammatory cytokines and oxidative damage. Nrf2 overexpression alleviated IR-induced lung injury and immunological responses. These data suggested that Nrf2 played a protective role against IR-induced lung injury and inflammation.

Acute lung injury and inflammation involve destruction of the lung structure and a cascade of inflammatory events, including the alterations of inflammatory cells and inflammatory cytokines (3–5). Activation of the Nrf2 signaling is a well-known mechanism that protects cells against radiation-induced oxidative stress (12, 13), but its role in RILI is not well-understood. Our data showed that alveolar septal thickness, structure damage, hemorrhage, and inflammatory cell infiltration were observed in sections of lung tissues after IR in both Nrf2-knockout and wild-type mice. These pathologic changes of IR-induced acute lung injury were much more serious in the Nrf2-knockout mice compared with the wild-type mice. Similarly, Traver et al. reported that Nrf2 loss promoted IR-induced alveolar type 2 cell loss and initiated a fibrotic phenotype (10). Inflammation is thought to play critical roles in RILI (15, 16, 17). Jiang et al. reported that 15 Gy of irradiation induced alveolar wall thickening and minimal interstitial edema in rat lung tissues, and increased serum levels of IL-1, IL-6, TNF-α, and IL-10 on day 3, 7, 14, and 28 after IR (2). IR induced inflammatory cytokines and chemokines, which caused infiltration of inflammatory cells such as macrophages and neutrophils (3, 18, 19). In turn, increased infiltration of inflammatory cells in lung tissues would promote the secretion of pro-inflammatory cytokines and the synthesis of ROS (3, 18, 20, 21). In this study, we found that IR promoted the infiltration of microphages and neutrophils in lung tissues, suggesting pathological manifestations of inflammation. Furthermore, Nrf2 deficiency increased IR-induced microphage and neutrophil infiltration in lung tissues. The inflammatory cell infiltration might cause consequent burst of inflammatory cytokines and result in aggravated lung injury. IR increased the serum levels of IL-6, MCP-1, IFN-γ, TNF, and IL-12p70, as well as, IL-10. Similar to inflammatory infiltration, loss of Nrf2 aggravated IR-induced increases of serum inflammatory cytokines IL-6, MCP-1, IFN-γ, TNF, and IL-12p70, but reduced the increase of IL-10. These data suggested that Nrf2 deficiency significantly aggravated IR-induced acute lung injury and inflammation, including destruction of the structure and pathological changes of pneumonia, infiltration of microphages and neutrophils, as well as, the imbalance of inflammatory cytokines.

IR induces the production of ROS and the excessive accumulation of ROS causes oxidative damage in lung tissues (7, 22, 23). ROS-induced oxidative damage in lung tissues might be one of the origin of inflammatory cell infiltration and imbalance of inflammatory cytokines. Our data showed that IR significantly increased oxidative damage in lung tissues of mice. As a consequence, the Nrf2 signaling pathway, an important antioxidative signaling cascade, was upregulated in lung tissues. Nrf2 deficiency aggravated IR-induced oxidative damage and reduced the upregulation of Nrf2 signaling cascade after IR. Therefore, we deduced that Nrf2 might have a protective role against RILI and inflammation. Previous studies indicated that upregulation of Nrf2 protected against RILI and lung fibrosis both *in vivo* and *in vitro* (12, 13, 24, 25). In our study, a Nrf2-overexpression mice model was established via *in vivo* transfection. As we expected, excess Nrf2 alleviated IR-induced infiltration of microphages and neutrophils in lung tissues, as well as, the serum levels of pro-inflammatory cytokines. The serum IL-10 levels were higher in Nrf2-overexpression group compared with vector-control mice after IR. In addition, Nrf2 overexpression promoted the upregulation of antioxidative signaling cascade after IR. As a consequence, IR-induced oxidative damage was reduced by Nrf2 overexpression. Hence, our data shows that Nrf2 had a protective role against IR-induced acute lung injury and inflammation through the reduction of oxidative damage in lung tissues through both Nrf2 deficiency and over-expression mice model.

We also test the levels of TGF-β1/Smads signaling pathway proteins in lung tissues of mice (Figure S4A). The results indicated that Nrf2 loss increased the protein levels of TGF-β1, and phosphorylated Smad2/3. Collagen content was indirectly determined by assessing the hydroxyproline levels in freshly obtained lung tissues. These results showed that Nrf2 deficiency significantly promoted IR-induced hydroxyproline in lung tissues (Figure S4B), and that Nrf2 overexpression significantly reduced IR-induced hydroxyproline levels (Figure S4C). Elizabeth et al. reported that Nrf2 deficiency reduced life span of mice administered thoracic irradiation (11). Taking together, our results indicated that Nrf2 might protect against RILI including oxidative-induced destruction of the lung tissues, inflammatory cell infiltration, imbalance between pro- and anti-inflammatory factors, and activation of pro-fibrotic signaling, the mechanism might be through the activation of anti-oxidative cascades. The limitation of this study is that the effects of Nrf2 were only evaluated in the early phase of RILI. A long-term study for several months to 1 year to identify the roles of Nrf2 in IR-induced lung fibrosis is required to further confirm our conclusions.

In summary, our study was the first to evaluate the protective role of Nrf2 against RILI and inflammation in both Nrf2-knockout and over-expressing mice model, and our data supported the concept that Nrf2 played a protective role against RILI and inflammation through the upregulation of antioxidative capacity and consequent alleviation of oxidative damage in lung tissues. Therefore, our findings suggest that antioxidative therapy might be a promising treatment for RILI.

## Author contributions

XT, YG, and CX: Conceptualization. XT, FW, and YL: Methodology. XT, FW, YL, SM, NZ, YS, CY, GT, and SL: Investigation. XT, FW, YL, and SM: Data analysis. XT: Writing-original draft. XT, YG, and CX: Writing-review & editing. CX: Supervision. CX and YG: Funding acquisition.

### Conflict of interest statement

The authors declare that the research was conducted in the absence of any commercial or financial relationships that could be construed as a potential conflict of interest.

## Abbreviations

RILI: Radiation-induced lung injury
Nrf2: Nuclear factor-erythroid 2-related factor 2
IR: Ionizing radiation
ROS: Reactive oxygen species
AREs: Antioxidant response elements
GPx1: Glutathione peroxidase-1
SOD: Superoxide dismutase
CAT: Catalase
MDA: Malondialdehyde
HO-1: Hemeoxygenase-1
NC: Non-instrumented control
MPO: Myeloperoxidase
8-OHdG: 8-Hydroxy-2′-deoxyguanosine
4-HNE: 4-Hydroxynonenal
CBA: Cytometric Bead Array
IL-6: interleukin-6
IL-10: Interleukin- 10
MCP-1: Monocyte chemoattractant protein-1
IFN-γ: interferon-γ
TNF: tumor Necrosis Factor
IL-12p70: Interleukin-12p70
TGF-β1: Transforming growth factor-β1
WT: Wild-type
KO: Knockout
Lv-Nfe2l2: Lentivirus carrying Nfe2l2
Lv-vector: Lentivirus carrying empty vector.
