# Supplementary material for: Protective Role of Nuclear Factor-Erythroid 2-Related Factor 2 Against Radiation-Induced Lung Injury and Inflammation
Source: Front Oncol. 2018 Nov 23;8:542. doi: 10.3389/fonc.2018.00542 (PMC6265406; doi:10.3389/fonc.2018.00542)
Supplement: Supplementary file 1 [file Data_Sheet_1.pdf]

Supplementary Materials

Protective Role of Nuclear Factor-Erythroid 2-Related Factor 2 against Radiation-Induced Lung Injury and Inflammation

Xiaoli Tian, Feng Wang, Yuan Luo, Shijing Ma, Nannan Zhang, Yingming Sun, Chengcheng You, Guiliang Tang, Shuying Li, Yan Gong, Conghua Xie*

*** Correspondence:** Conghua Xie, chxie_65@whu.edu.cn

Supplementary Figures

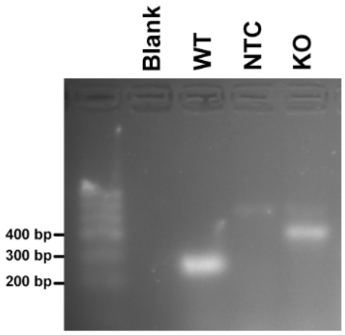

Fig. S1. Validation of Nrf2-knockout mice(Nfe2l2$^{-/-}$). Mice were validated via standard PCR according to the instruction of Jackson Laboratory (https://www2.jax.org/protocolsdb/f?p=116:5:0::NO:5:P5_MASTER_PROTOCOL_ID,P5_JRS_CODE:7474,017009). Mutant = ~400 bp, Heterozygote = ~400 bp and 262 bp, Wild type = 262 bp; (WT: wild-type mice; KO: Nrf2 knockout mice; NTC: PCR reaction mixture control without DNA template)

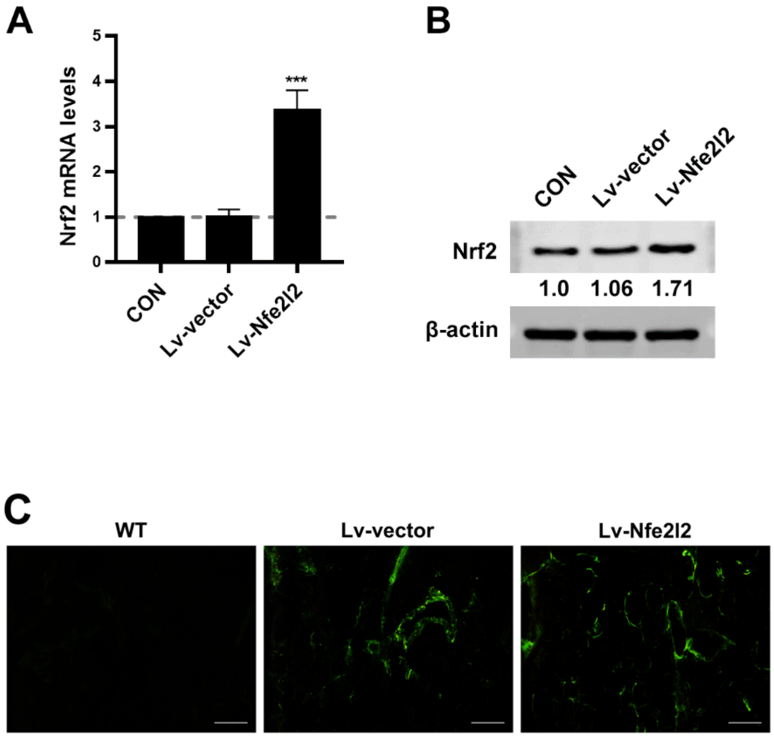

Fig. S2. Validation of *in vivo* transfection of Nrf2 in lung tissues of mice. (A) qPCR was used to validate the overexpression of Nrf2 mRNA in lung tissues. The primers of Nrf2 are shown as follows: F: CTGGCTGATACTACCGCTGTTC, R: AGGTGGGATTTGAGTCTAAGGAG. (B) Immunobloting was used to validate the overexpression of Nrf2 proteins in lung tissues. n = 5. *** represents P<0.001. Every experiment was repeated for 3 times. CON: Untreated-control wild-type mice. Lv-vector: wild-type mice transfected with lentivirus carrying empty vector. Lv-Nfe2l2: wild-type mice transfected with lentivirus carrying Nfe2l2. (C) The efficiencies of lentiviral-mediated transfection were determined by fluorescent imaging of lung tissues. Scale bar: 100 μm.

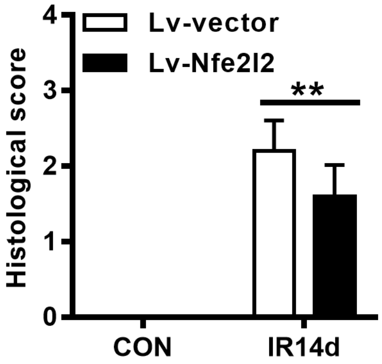

Fig. S3. Nrf2 overexpression alleviates radiation-induced lung injury and inflammation.
Histology scores of HE staning. ** represents P<0.01.

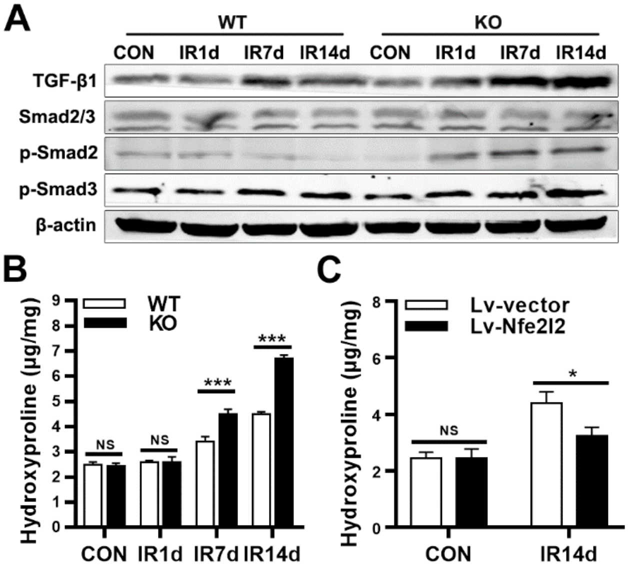

Fig. S4. Alterations of TGF-β1/Smads signaling and hydroxyproline levels. (A) Nrf2 knockout promoted IR-induced activation of TGF- β 1/Smads signaling. (B) Nrf2 knockout aggravated IR-induced hydroxyproline deposition in lung tissues. n = 6. (C) Nrf2 overexpression inhibited IR-induced hydroxyproline deposition in lung tissues. n = 5. ^{NS} represents no statistical difference (*P*>0.05). * represents *P*<0.05. ** represents *P*<0.01. *** represents *P*<0.001. Every experiment was repeated for 3 times. The data was shown as *mean* ± *SD*.
